# Preliminary Bioequivalence of an Oral Pimobendan Solution Formulation with Reference Solution Formulation in Beagle Dogs

**DOI:** 10.3390/vetsci9030141

**Published:** 2022-03-17

**Authors:** Nakkawee Saengklub, Tussapon Boonyarattanasoonthorn, Anusak Kijtawornrat, Doungdaw Chantasart

**Affiliations:** 1 Department of Physiology, Faculty of Pharmacy, Mahidol University, Bangkok 10400, Thailand; nakkawee.sae@mahidol.ac.th; 2 Center of Innovative Pharmacy, Faculty of Pharmacy, Mahidol University, Bangkok 10400, Thailand; 3 Department of Physiology, Faculty of Veterinary Science, Chulalongkorn University, Bangkok 10330, Thailand; tussapon.b@chula.ac.th; 4 Chulalongkorn University Laboratory Animal Center, Chulalongkorn University, Bangkok 10330, Thailand; 5 Department of Pharmacy, Faculty of Pharmacy, Mahidol University, Bangkok 10400, Thailand

**Keywords:** bioequivalence, cardiac function, dog, echocardiography, ODMP, oral, pimobendan, pharmacodynamics, pharmacokinetics, solution

## Abstract

Oral capsule and tablet formulations of pimobendan are widely used but may present difficulties for accurately dosing small patients. This study aimed to compare the pharmacokinetic (PK) characteristics, bioequivalence, and cardiovascular effects of a custom-made oral pimobendan solution (PS) formulation compared to a reference solution (RS) formulation in conscious, healthy dogs. A randomized crossover design was performed on dogs that received RS and PS formulations at a dose of 0.3 mg/kg. Blood samples were collected at 0, 0.083, 0.167, 0.25, 0.5, 0.75, 1, 1.5, 2, 3, 4, 8, and 24 h after oral administration for PK analysis; bioequivalence was also calculated. Echocardiography was also performed to assess the cardiovascular effects. The results revealed that the plasma concentrations of pimobendan and o-desmethyl-pimobendan (active metabolite) in the case of both formulations were comparable. The relative ratios of geometric mean concentrations for all significant parameters of PK were within a range of 80–125%, indicating bioequivalence. In addition, both formulations increased cardiac contraction significantly when compared with the baseline, and no differences in cardiac contractility were detected between the formulations. The PS formulation can be used as alternative to the RS formulation for the management of congestive heart disease because of the bioequivalence between the two formulations.

## 1. Introduction

Pimobendan, a benzimidazole–pyridazinone derivative, is a phosphodiesterase III (PDE-3) inhibitor as well as a calcium sensitizer [1]. Its unique properties lead to an increase in cardiac contractions as well as promoting vascular relaxation, mainly by increasing the intracellular cAMP in cardiac myocytes and vascular smooth muscle cells. In addition, the increased myocardial contraction is owing to the increased affinity of troponin C to cytosolic calcium. Pimobendan has been widely used in veterinary medicine for the management of congestive heart failure (CHF) caused by either dilated cardiomyopathy (DCM) [2] or myxomatous mitral valve disease (MMVD) [3]. Recent publications have supported the use of pimobendan in asymptomatic MMVD stage B2 [4,5] and have led the American College of Veterinary Internal Medicine to publish new guidelines for the diagnosis and treatment of MMVD in dogs [6].

According to previous studies in dogs, the oral bioavailability of the pimobendan chewable tablet is about 70% [1,7]. After absorption, pimobendan is oxidatively demethylated into the active metabolite, o-desmethyl-pimobendan (ODMP), and is conjugated with sulfate or glucuronic acid, which is mainly excreted via feces [8]. Protein binding is >90% and the half-lives of pimobendan and ODMP are 0.5 h and 2 h, while the steady-state volume of distribution (V_d_) of pimobendan is 2.6 L/kg. The clearance of pimobendan is about 90 mL/min/kg, and the plasma levels of pimobendan and ODMP are below quantifiable levels by 4 and 8 h after oral administration, respectively. In dogs, pimobendan exerts positive inotropic and lusitropic properties 1 h after oral administration, while ODMP exerts cardiovascular effects up to 8–12 h after administration [9].

The original pimobendan formulation has been supplied in the form of a hard capsule with an orange/white color, followed by an easily chewed and swallowed tablet, and it has caused difficulty in accurately dosing small patients. Currently, oral pimobendan solution (Vetmedin, Boehringer Ingelheim Vetmedica GmbH, Ingelheim, Germany) is available in a limited number of countries (e.g., Australia). In addition, the original pimobendan solution is supplied at a concentration of 3.5 mg/mL, which is still difficult to accurately administer to small patients, such as cats and dogs (e.g., Chihuahua, Pomeranian, Russian Toy). Therefore, a custom-made pimobendan solution may serve as an alternative to the currently existing, original solution of this drug, which is not available in most countries. This study was designed to compare the pharmacokinetic characteristics, bioequivalence, and cardiovascular effects, as assessed by echocardiography, of pimobendan and ODMP after single, oral administration of our pimobendan solution formulation (2.5 mg/mL) to the reference (original) solution formulation (3.5 mg/mL) in conscious, healthy beagle dogs. The hypothesis is that the new oral pimobendan solution formulation would be a bioequivalent to the reference solution formulation.

## 2. Materials and Methods

### 2.1. Pimobendan Oral Solution Formulation

The pimobendan oral solution (PS) formulation was prepared to a concentration of 2.5 mg/mL of pimobendan (Hangzhou Hyper Chemicals Limited, Zhejiang, China). The drug powder was dissolved in a suitable solvent system. Glycerin (Namsiang, Bangkok, Thailand) was used as the major component in the solvent system. The drug content and chemical stability of the pimobendan oral solution was analyzed using a high-performance liquid chromatography (HPLC) method that was slightly modified from that of Yata and colleagues [10]. Briefly, the HPLC system consisted of a pump, a degasser, an autosampler, a UV–Vis detector (Shimadzu Corporation, Kyoto, Japan) and a Hypersil^®^ BDS C18 column with a guard column (5 μm particle size, 150 mm × 4.6 mm; Thermo Fisher Scientific Inc., Waltham, MA, USA). The mobile phase consisted of 0.1% formic acid in water (solvent A) and 0.1% formic acid in acetonitrile (solvent B) run at 1 mL/min. The gradient started at 2% solvent B, was held for 3 min (0–3 min), ramped to 75% solvent B at 3–8 min, and then held for 2 min (8–10 min). Solvent B returned to 2% at 10–12 min. The assay was run at room temperature. The injection volume was 10 μL. The detection wavelength was 332 nm. The retention time was at 6.9 min. A good linearity of standard curves of pimobendan in the mobile phase was obtained in the range of 1.25–20 μg/mL (*R*^2^ = 0.999). The level of detection and limit of quantitation for pimobendan were 0.0857 and 0.2597 μg/mL, respectively. Fresh preparations were used for the pharmacokinetic and pharmacodynamic studies.

### 2.2. Animals

The animal procedures were approved by the Chulalongkorn University Animal Care and Use Committee (IACUC) at Chulalongkorn University Laboratory Animal Center (CULAC Animal Use Protocol number 2173038). The procedures were conducted in accordance with the Animals for Scientific Purposes Act, B.E. 2558 (A.D. 2015), and the *Guide for the Care and Use of Laboratory Animals* [11]. Four male, adult beagles (*Canis lupus familiaris*), weighing 12–15 kg, were included in the current study and were housed in groups at a temperature between 21 °C and 23 °C, room humidity between 30% and 70%, and a 12:12 h light/dark cycle (light on between 6:00 a.m. and 6:00 p.m.). Dogs were fed once a day and were provided unlimited access to drinking water. Animals were fasted 12 h prior to the administration of either the PS formulation or the reference solution (RS). All animals were fed 4 h after administration to reduce interference from diet in the outcome of the study. Physical examination, a full blood count, a blood chemistry panel (i.e., creatinine, alkaline phosphatase, alanine aminotransferase, aspartate aminotransferase, blood urea nitrogen, total protein, albumin, globulin, and glucose), and a lead II electrocardiogram (ECG), were obtained to assess their health before conducting the study. Upon initial evaluation, animals were excluded from the experiment if there were signs of illness from systemic or heart disease.

### 2.3. Drug Administration and Blood Sample Acquisition

The current study was a randomized crossover design. Each dog was given both the pimobendan oral solution (2.5 mg/mL) and the reference solution (3.5 mg/mL) (Vetmedin, Boehringer Ingelheim Vetmedica GmbH, Ingelheim, Germany) in a crossover fashion, separated by a 2-week washout period. The half-lives of pimobendan and its active metabolite, ODMP, in dogs were reported to be about 0.9 h and 1.6 h [7]. The washout period was in accordance with the recommendation provided by EMEA, which indicates that it should be at least five times the terminal half-life [12]. The drug administration order was randomized to be given to the dogs at 8:00 and 9:00. The solution was administered orally at a dose of 0.3 mg/kg. To ensure that the dog had all medications, ten milliliters of RO-UV treated water was given using a syringe after solution administration.

Blood samples (1 mL) were collected from the saphenous vein at baseline (before dosing) and 0.083, 0.167, 0.25, 0.5, 0.75, 1, 1.5, 2, 3, 4, 8, and 24 h following administration. The samples were processed as previously described [13]. Briefly, samples of venous blood were placed in lithium heparin tubes. Then, the samples were centrifuged within 1 h after collection to separate plasma at 5000× *g* at 4 °C for a total of 10 min. The plasma samples were kept at −20 °C for further analyses.

After the PK data was collected from all dogs, the pharmacodynamic study was assessed in a different study in which all dogs were randomized to receive both pimobendan solution formulations again in a crossover fashion with a 2-week washout period.

### 2.4. Drug Analysis by Liquid Chromatography Tandem Mass Spectrometry

Plasma samples were thawed at room temperature and processed as previously described [13]. Briefly, each sample (50 μL) was mixed with absolute methanol (200 μL) containing the glycyrrhizin (100 ng/mL) as an internal standard. Then, the mixtures were centrifuged at 10,000× *g* for a total of 10 min to collect supernatant (10 μL). The supernatant was injected into the liquid chromatography tandem mass spectrometry system as previously described by Pichayapaiboon and colleagues [13]. The liquid chromatography tandem mass spectrometry module used in the current study was the Nexera ultra high-performance liquid chromatography and the 8060 triple quadrupole mass spectrometer (Shimadzu Co., Ltd., Kyoto, Japan). The stationary phase was the Synergi Fusion-RP C18 column (Phenomenex, Inc., Torrance, CA, USA). During analyses, the oven temperature was maintained at 40 °C.

In the current study, 0.2% formic acid in water (solvent C) and absolute methanol (solvent D) were used as a mobile phase. The gradient started at 10% solvent D and was held for 0.5 min (0–0.5 min), and then ramped to 90% solvent D at 0.5–1.5 min and was held for 1.5 min (1.5–3.0 min). The solvent D returned to 10% at 3.0–4.0 min and was held for 1 min (4.0–5.0 min). The retention times of pimobendan, ODMP, and the internal standard were 2.12, 1.58, and 2.05 min, respectively, and the mass-to-charge ratios of each compound were 335/319, 321.10/305.05, and 821.25/350.90 *m*/*z*, respectively. The lower limit for detection for both pimobendan and ODMP was 0.09 μg/L. The good linearity of the standard curves for pimobendan and ODMP in the mobile phase was obtained in the range of 0.09–100 μg/L (*R*^2^ > 0.99) and 0.09–200 μg/L (*R*^2^ > 0.99), respectively. The accuracy ranged from 92.70–100.52% and 93.10–109.40% for pimobendan and ODMP, respectively. The precision (% CV) ranged from 4.04–8.96% for pimobendan and from 4.78–9.43% for ODMP. The intra-day and inter-day precision and accuracy were determined at concentrations from 1 to 100 µg/L for pimobendan and from 1–200 µg/L for ODMP. The percent recoveries of both compounds were greater than 70%.

### 2.5. Pharmacokinetics

PK solution software version 2.0 (Summit Research Services, Montrose, CO, USA) was used for PK analysis and the noncompartmental model was utilized. For each dog, plasma concentration–time curves were used to obtain C_max_ and T_max_. The AUC_0__–t_ was calculated from the trapezoidal rule. The equation AUC_0__–inf_ = AUC_0__–t_ + (C_t_/k_el_) was used to extrapolate from time zero to time infinity, in which the C_t_ is the last-observed plasma concentration after dosing. The k_el_ is the elimination rate constant, which is calculated using the log-linear slope of the terminal phase of the concentration-time curve. Mean residence time (MRT) was determined by AUMC_0__–inf_/AUC_0__–inf_. The AUMC_0__–inf_ is the area under the first moment concentration–time curve. The total clearance (CL) was equal to dose/AUC_0__–inf_. The volume of distribution (V_d_) was calculated as CL/k_el_. The terminal elimination half-life was equal to 0.693/k_el_.

### 2.6. Effects of Drugs on Cardiac Function

In a separate study, dogs were given both formulations, similar to the PK study, to determine the effects of the drug in both formulations on cardiac function. Echocardiography was performed in all dogs at baseline (before dosing), and 0.083, 0.167, 0.25, 0.5, 0.75, 1, 1.5, 2, 3, 4, 6, 8, and 12 h following administration, without sedation, using an ultrasound unit (M-9, Mindray, Shenzhen, China) equipped with phased array transducers (1–5 MHz). The process of echocardiographic examination was conducted in compliance with the *Guidelines for the American Society of Echocardiography* [14]. All echocardiographic examinations were performed by a veterinarian (AK) who specializes in cardiac ultrasound, blinded to the study. The left atrial to aortic root ratio (LA/Ao), the left ventricular internal diameter diastole normalized by body weight (LVIDDN), and the left ventricular internal diameter systole normalized by body weight (LVIDSN) were obtained via the right parasternal short-axis view and M-mode, as previously described [5]. End-diastolic volume (EDV), end-systolic volume (ESV), and ejection fraction (EF) were determined using a modified Simpson’s Method of Discs [15] on the right parasternal, long-axis, 4-chamber view. EDV and ESV were indexed to body surface area (i.e., EDVI and ESVI, respectively). The stroke volume (SV) and cardiac output (CO) were calculated, while the heart rate (HR) was obtained from the ECG and was simultaneously recorded with the echocardiography. The intraobserver and interobserver variabilities of the echocardiographic parameters of the authors were as performed and published previously [16].

### 2.7. Bioequivalence and Data Analysis

Pharmacokinetic parameters were presented as mean ± standard deviation (SD), while pharmacodynamic data (i.e., effects of drugs on cardiac function) were presented as mean ± standard error of the mean (SEM). In the section on pharmacokinetics, the ratio of the geometric means of the PK variable values for the pimobendan and ODMP, of both the pimobendan oral solution formulation and the reference formulation, were calculated. Analysis of variance (ANOVA) and 90% confidence interval (CI) were determined, as previously described [17]. Briefly, ANOVA was performed on the PK parameters using the linear mixed-effects model, including animals within the sequence as a random effect, and the sequence, period, and formulation as fixed effects. The two formulations were bioequivalent if 90% of the CI fell within the limits of 80 to 125% [12,18].

For the echocardiographic parameters, a normality test was performed using the Shapiro–Wilk test. Two-way ANOVA with repeated measures was used to analyze the differences between the groups (pimobendan oral solution formulation vs. reference formulation), and within groups between timepoints. The IBM^®^ SPSS^®^ software platform licensed by Chulalongkorn University was used for all statistical analysis and a *p*-value < 0.05 indicated statistical significance.

## 3. Results

The analysis of drug content and chemical stability of the developed pimobendan oral solution revealed that the amounts of pimobendan in the pimobendan oral solution, in amber plastic bottles, were greater than 90%, and no degraded compound was found after storage in a refrigerator (2–8 °C), at room temperature (30 °C) and at 40 °C for 120 days.

**Figure 1:**
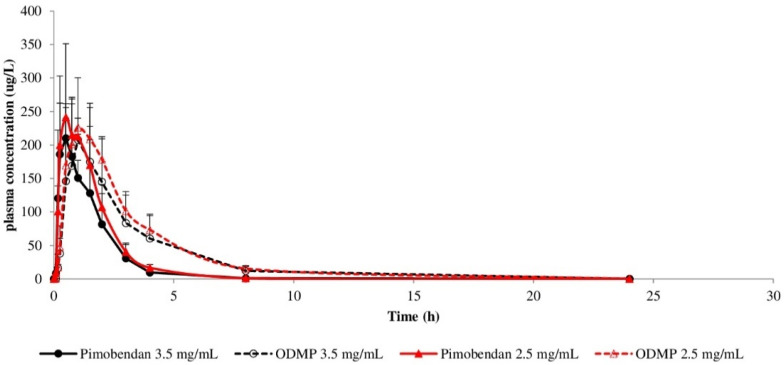
Time course of plasma pimobendan and ODMP concentrations (mean ± SD) in dogs. The pimobendan oral solution formulation (2.5 mg/mL) and reference solution formulation (3.5 mg/mL) were administered at a dose of 0.3 mg/kg in a crossover fashion (n = 4 for each formulation).

In the current study, the clinical examination, hematology values, and blood chemistry tests of all animals before starting the in vivo experimental study were within normal limits. All dogs tolerated both formulations well, with no obvious clinical signs of gastrointestinal discomfort or other undesirable effects observed after administration. The plasma levels of pimobendan and ODMP after oral administration of both pimobendan formulations at 0.3 mg/kg were plotted versus time as mean ± SD (Figure 1). In general, plasma concentrations of pimobendan and ODMP of both formulations were comparable. The mean plasma concentrations of pimobendan of both formulations peaked at 40–45 min after administration, while the plasma concentration of ODMP gradually increased from 0 µg/L at baseline and reached its maximal plasma concentration within 60–69 min, for both formulations, after administration of the parent drug. After pimobendan and ODMP reached their maximal plasma concentrations, both pimobendan and ODMP began to decline slowly until the end of the experiment; pimobendan declined faster than ODMP.

The pharmacokinetic parameters of both pimobendan formulations that were calculated using non-compartment methods are shown in Table 1. According to the ANOVA presentation of the calculation of bioequivalence, no statistically significant difference between the formulations was detected using ANOVA for PK parameters.

The inotropic properties of the left ventricle, in response to both formulations, were assessed using the parameters obtained from the echocardiography (Table 2). The oral administration of pimobendan had no effect on LA/Ao, LVIDDN, EDVI, or HR. LVIDSN decreased after oral administration, differed significantly from baseline at 0.5 h after oral administration (*p* < 0.05), and remained significantly different until 6 h after oral administration. The ESVI and EF of the RS formulation and PS formulation at baseline were 22.1 ± 1.8 vs. 22.1 ± 1.6 and 66.6 ± 0.8% vs. 64.8 ± 0.5%, respectively. Compared with the baseline, ESVI decreased while EF increased in response to oral administration of pimobendan and became significant at 0.25 h (*p* < 0.05). Both ESVI and EF remained significant until 6 h after oral administration. Additionally, the SV of the RS formulation and PS formulation at baseline were 24.4 ± 1.7 mL vs. 23.0 ± 1.7 mL, increased significantly at 1.5 h after oral administration (*p* < 0.05), and remained significantly increased from baseline until 3 h. The cardiac output, a product of HR and SV, tended to increase at 1.5 h and 2 h. Interestingly, there was no significant difference between formulations at any measured timepoint.

## 4. Discussion

This study was conducted to compare the PK profiles, bioequivalences, and cardiac effects of a newly developed pimobendan oral solution formulation to a reference formulation in healthy, male beagle dogs. PS showed a complete PK bioequivalence and similar cardiac effects to the reference solution.

The developed pimobendan oral solution, using glycerin as the major component in a solvent system, was chosen for the following reasons: (i) glycerin is a colorless, odorless, viscous liquid that is sweet-tasting and nontoxic; (ii) the homogeneous system of the pimobendan oral solution (2.5 mg/mL) can be prepared in the solvent system with glycerin as the major component; and (iii) dogs did not refuse the developed pimobendan oral solution. In addition, the chemical stability of the developed pimobendan oral solution was stable in amber plastic bottles in a refrigerator (2–8 °C), at room temperature (30 °C), and at 40 °C for 120 days.

In veterinary medicine, most dogs presenting with CHF due to MMVD are small-breed dogs; therefore, the oral administration of pimobendan may be challenging in the countries where chewable tablets or capsules are the only available form. Furthermore, in countries where the pimobendan reference solution (3.5 mg/mL) is available, the concentration is still high, which makes the dosing accuracy questionable. For these scenarios, an oral pimobendan solution at a lower concentration may provide an alternative option for small-breed dogs, or even cats.

This study demonstrated that the oral administration of pimobendan solution of both formulations at a dose of 0.3 mg/kg can achieve a plasma level of pimobendan and ODMP in healthy dogs, which is higher than those provided in the prescribing information [8]. The authors are aware of the disparity among the PK profiles of pimobendan reported in previous publications [1,7]. For example, Bell and colleagues gave oral capsules of pimobendan at a dose of 0.25 mg/kg to healthy dogs, which led to a C_max_ of 38.1 ± 18.3 ng/mL [1], while Yata and colleagues gave a nonaqueous pimobendan solution at a dose of 0.27 mg/kg to healthy dogs and resulted in a C_max_ of 18.6 ng/mL [7]. In the current study, the C_max_ for both the PS and reference solution formulations were 269.6 ± 50.4 ng/mL and 240.9 ± 15.6 ng/mL, respectively, which are higher than those of previous studies [1,7,8]. This may be due mainly to the differences in the dosages given to the dogs. It is also known that differences in the design of experiments (e.g., analytical method, dosage, and age of subjects) or the effects of different formulations may lead to such discrepancies [19]. Although the C_max_ values in the different studies are different, the clearance of pimobendan in the current study is similar to previous reports [8].

Furthermore, the findings of the current PK study indicate that the pimobendan of both the PS and RS formulations appears to be more rapidly absorbed (0.75 h and 0.67 h, respectively) when compared with a previous study (1.1 h) [7]. In addition, the pimobendan of both the PS and RS formulations was more rapidly converted into its metabolite ODMP (1.15 h and 1.10 h, respectively) when compared with a previous study (1.3 h) [7]. Both of these findings appear to be different from those listed in the package insert. The product label reports that oral pimobendan (0.25 mg/kg) administration in dogs results in T_max_ of pimobendan and ODMP of 2 and 3 h after administration [8]. It has been known that oral pimobendan undergoes oxidation in the process of first-pass hepatic metabolism and is changed into ODMP, which shows that the PDE-3i effect is more potent than the parent compound [20,21]. Therefore, in our PD study, pimobendan contributes to the first phase of cardiac effects, while the ODMP may contribute to the later phase of cardiac effects.

In the current study, the effect of pimobendan on heart function was determined using echocardiographic parameters. In response to oral pimobendan, both EF and SV increased, while LVIDSN and ESVI decreased, suggesting that pimobendan and ODMP increase cardiac contractility. The increase in cardiac inotrope in the present study could be explained by the sensitization of troponin C to calcium in cytosol and the inhibition of PDE-3 [22]. This result is also in accordance with our previous studies [13] and those of others [23,24], in which cardiac contractility increased significantly when pimobendan was given to anesthetized dogs.

The potential limitations of the current study are the small sample size (n = 4) and the fact that only male beagle dogs were used. We are aware that the recommended sample size of a bioequivalence test by the EMEA and FDA is 12 [12,18]. The sample size of the current study was limited to 4 due to the power analysis of previous publications yielding enough power [17]. In addition, most of the vital PK and PD parameters in the current study showed low variations due to the ages of the dogs being similar, as well as their body weights. Another potential limitation is the lack of a control group. The main purpose of the current study was to compare the two formulations on PK and PD in healthy dogs; therefore, the statistical analysis set up was designed according to the main purpose, in which a control group was not included. Thus, the lack of a control group may not interfere with the outcome of the study.

## 5. Conclusions

The results of the current study indicate that pimobendan at therapeutic doses can be delivered via our newly developed solution or reference solution. The major advantages of our PS formulation over the reference formulation in animals are convenience and accurate dosing for small patients. It can be concluded that this PS can be used as an alternative to the reference solution for the management of congestive heart disease because of the bioequivalence between the two formulations. However, further studies are needed to confirm the complete bioequivalence in the case of sex differences, as well as with more subjects.

## Figures and Tables

**Table 1:** Comparison of pharmacokinetic parameters between pimobendan oral solution formulation (2.5 mg/mL) and reference solution formulation (3.5 mg/mL) after 0.3 mg/kg oral administration in healthy beagle dogs (n = 4 for each formulation).

PK Parameters	Pimobendan				O-Desmethyl-Pimobendan (ODMP)			
	Reference	PS	Ratio ^#^	90% CI ^#^	Reference	PS	Ratio ^#^	90% CI ^#^
C_max_ (µg/L)	240.9 ± 15.6	269.6 ± 50.4	89.1	80.9–97.3	241.8 ± 64.4	253.3 ± 8.7	95.5	85.1–107.2
T_max_ (h)	0.67 ± 0.29	0.75 ± 0.29	88.0	66.0–115.2	1.10 ± 0.36	1.15 ± 0.40	95.7	75.3–119.6
AUC_0–24_ (µg.h/L)	414.4 ± 98.2	439.5 ± 55.5	95.5	83.2–109.7	880.3 ± 82.4	818.1 ± 79.7	107.2	97.3–115.5
AUC_0__–inf_ (µg.h/L)	415.5 ± 98.2	440.3 ± 55.4	95.5	82.6–108.9	880.6 ± 82.4	818.4 ± 79.7	107.2	98.3–115.5
MRT (h)	8.27 ± 0.25	8.50 ± 0.87	97.8	93.3–104.7	5.07 ± 0.68	5.00 ± 0.30	100.0	93.4–107.2
V_d_ (L/kg)	43.9 ± 10.4	43.2 ± 9.3	100.0	82.9–119.9	9.23 ± 0.72	8.67 ± 1.15	107.1	97.6–117.5
CL (L/h/kg)	5.80 ± 1.59	5.74 ± 0.71	100.0	85.9–115.9	2.86 ± 0.28	2.92 ± 0.39	97.6	89.2–104.8
Half-life (h)	4.72 ± 0.19	4.68 ± 0.53	100.0	93.2–107.1	2.25 ± 0.22	2.21 ± 0.10	102.3	97.7–125.7

^#^ Based on analysis of variance with a linear mixed-effects model that contained effects for sequence, subject nested within sequence, period, and formulation for logarithmically transformed data. PK parameters are expressed as mean ± SD; C_max_: maximum plasma concentration; T_max_: time to reach C_max_; AUC_0–24_: area under the plasma concentration–time curve from time 0–24 h; AUC_0–inf_: area under the plasma concentration–time curve from time 0–infinity; MRT: mean resident time; V_d_: volume of distribution; CL: clearance.

**Table 2:** Comparison of the cardiac effects of pimobendan (0.3 mg/kg) for both reference solution formulation (3.5 mg/mL) and pimobendan oral solution formulation (2.5 mg/mL) on the echocardiographic parameters in healthy beagle dogs (n = 4 for each formulation).

Timepoints	Formulations	LA/Ao	LVIDDN (cm)	LVIDSN (cm)	EDVI	ESVI	EF (%)	SV (mL)	CO (L/min)	HR (bpm)
**Baseline**	Reference (3.5 mg/mL)	1.4 ± 0.09	1.4 ± 0.04	0.9 ± 0.03	65.8 ± 4.3	22.1 ± 1.8	66.6 ± 0.8	24.4 ± 1.7	2.5 ± 0.44	102 ± 14.6
	PS (2.5 mg/mL)	1.3 ± 0.05	1.4 ± 0.04	0.9 ± 0.03	63.4 ± 4.4	22.1 ± 1.6	64.8 ± 0.5	23.0 ± 1.7	2.4 ± 0.13	107 ± 4.9
**0.083 h**	Reference (3.5 mg/mL)	1.3 ± 0.04	1.4 ± 0.02	0.9 ± 0.01	60.1 ± 2.1	20.6 ± 0.8	65.6 ± 0.4	22.0 ± 0.7	2.2 ± 0.27	98 ± 9.3
	PS (2.5 mg/mL)	1.3 ± 0.09	1.4 ± 0.05	0.8 ± 0.03	59.6 ± 5.7	19.9 ± 1.8	66.6 ± 0.3	22.0 ± 2.0	2.1 ± 0.14	96 ± 4.4
**0.167 h**	Reference (3.5 mg/mL)	1.3 ± 0.04	1.4 ± 0.05	0.8 ± 0.05	63.9 ± 5.6	19.7 ± 2.5	69.3 ± 1.9	24.1 ± 1.9	2.4 ± 0.18	97 ± 9.0
	PS (2.5 mg/mL)	1.3 ± 0.07	1.4 ± 0.03	0.9 ± 0.02	60.8 ± 3.4	19.9 ± 1.0	67.2 ± 0.5	22.7 ± 1.2	1.9 ± 0.12	83 ± 4.1
**0.25 h**	Reference (3.5 mg/mL)	1.3 ± 0.05	1.4 ± 0.05	0.8 ± 0.02	57.7 ± 5.8	17.1 ± 1.1 *	70.0 ± 1.1 *	22.5 ± 2.4	1.8 ± 0.31	91 ± 13.4
	PS (2.5 mg/mL)	1.2 ± 0.05	1.4 ± 0.03	0.8 ± 0.01	60.7 ± 3.2	17.9 ± 0.7 *	70.4 ± 1.4	23.8 ± 1.3	1.9 ± 0.20	82 ± 8.9
**0.50 h**	Reference (3.5 mg/mL)	1.2 ± 0.06	1.4 ± 0.07	0.7 ± 0.03 *	61.9 ± 7.4	12.6 ± 1.4 *	79.2 ± 1.9 *	27.2 ± 3.1	2.6 ± 0.45	97 ± 14.5
	PS (2.5 mg/mL)	1.3 ± 0.05	1.4 ± 0.06	0.7 ± 0.04 *	58.1 ± 6.0	13.3 ± 1.9 *	77.0 ± 2.4 *	24.7 ± 2.4	2.3 ± 0.24	95 ± 9.1
**0.75 h**	Reference (3.5 mg/mL)	1.3 ± 0.05	1.4 ± 0.04	0.7 ± 0.03 *	59.7 ± 3.8	11.3 ± 1.4 *	81.3 ± 1.8 *	26.9 ± 1.6	2.5 ± 0.19	95 ± 6.6
	PS (2.5 mg/mL)	1.3 ± 0.03	1.3 ± 0.03	0.7 ± 0.03 *	55.2 ± 2.9	10.8 ± 1.1 *	80.3 ± 2.5 *	24.6 ± 1.6	2.2 ± 0.11	92 ± 7.6
**1 h**	Reference (3.5 mg/mL)	1.2 ± 0.06	1.3 ± 0.05	0.6 ± 0.02 *	53.7 ± 4.8	7.5 ± 0.6 *	86.0 ± 1.0 *	25.6 ± 2.1	2.4 ± 0.15	98 ± 13.0
	PS (2.5 mg/mL)	1.2 ± 0.03	1.3 ± 0.07	0.6 ± 0.05 *	55.1 ± 7.2	8.1 ± 1.7 *	85.7 ± 1.5 *	26.0 ± 2.8	2.6 ± 0.25	103 ± 11.7
**1.5 h**	Reference (3.5 mg/mL)	1.2 ± 0.05	1.3 ± 0.04	0.5 ± 0.01 *	53.6 ± 3.7	5.8 ± 0.3 *	89.0 ± 0.3 *	26.5 ± 1.6 *	3.0 ± 0.22	114 ± 11.9
	PS (2.5 mg/mL)	1.3 ± 0.04	1.4 ± 0.04	0.5 ± 0.01 *	57.3 ± 4.2	6.0 ± 0.3 *	89.5 ± 0.4 *	29.2 ± 1.3 *	2.7 ± 0.41	97 ± 12.6
**2 h**	Reference (3.5 mg/mL)	1.3 ± 0.10	1.4 ± 0.05	0.5 ± 0.02 *	56.7 ± 5.4	4.9 ± 0.6 *	91.3 ± 0.4 *	28.6 ± 2.1 *	2.7 ± 0.29	108 ± 18.4
	PS (2.5 mg/mL)	1.2 ± 0.05	1.4 ± 0.05	0.5 ± 0.03 *	58.5 ± 5.1	4.8 ± 0.7 *	91.9 ± 0.5 *	29.8 ± 2.1 *	2.9 ± 0.31	100 ± 12.7
**3 h**	Reference (3.5 mg/mL)	1.2 ± 0.04	1.4 ± 0.05	0.6 ± 0.04 *	55.1 ± 5.7	7.6 ± 1.4 *	86.4 ± 1.1 *	26.4 ± 2.3 *	2.5 ± 0.25	99 ± 14.1
	PS (2.5 mg/mL)	1.3 ± 0.02	1.4 ± 0.03	0.6 ± 0.02 *	59.6 ± 3.1	8.5 ± 0.6 *	85.6 ± 1.3 *	28.4 ± 1.6 *	2.6 ± 0.16	93 ± 9.7
**4 h**	Reference (3.5 mg/mL)	1.2 ± 0.05	1.3 ± 0.05	0.7 ± 0.09 *	52.0 ± 5.0	9.0 ± 1.2 *	82.8 ± 0.6 *	23.8 ± 1.6	2.4 ± 0.43	117 ± 18.7
	PS (2.5 mg/mL)	1.2 ± 0.03	1.4 ± 0.03	0.7 ± 0.02 *	57.7 ± 2.8	10.6 ± 0.9 *	81.6 ± 0.8 *	26.2 ± 1.1	2.6 ± 0.21	98 ± 6.2
**6 h**	Reference (3.5 mg/mL)	1.3 ± 0.14	1.4 ± 0.04	0.8 ± 0.03 *	59.7 ± 4.5	15.3 ± 1.5 *	74.4 ± 0.8 *	24.7 ± 1.5	2.5 ± 0.39	101 ± 13.2
	PS (2.5 mg/mL)	1.2 ± 0.09	1.3 ± 0.06	0.7 ± 0.03 *	56.0 ± 6.1	13.3 ± 1.4 *	76.0 ± 1.9 *	23.7 ± 2.6	2.2 ± 0.31	94 ± 12.7
**8 h**	Reference (3.5 mg/mL)	1.3 ± 0.04	1.4 ± 0.03	0.9 ± 0.03	62.0 ± 3.0	20.2 ± 1.4	67.5 ± 1.2	23.2 ± 1.0	2.3 ± 0.19	98 ± 8.3
	PS (2.5 mg/mL)	1.3 ± 0.07	1.4 ± 0.04	0.8 ± 0.02	60.2 ± 4.5	19.9 ± 1.4	66.9 ± 0.4	22.4 ± 1.5	2.0 ± 0.13	89 ± 3.6
**12 h**	Reference (3.5 mg/mL)	1.3 ± 0.06	1.4 ± 0.03	0.9 ± 0.02	63.0 ± 3.0	21.4 ± 1.3	66.1 ± 0.5	23.2 ± 1.1	2.3 ± 0.35	100 ± 11.5
	PS (2.5 mg/mL)	1.3 ± 0.07	1.4 ± 0.04	0.9 ± 0.03	61.5 ± 5.0	21.0 ± 1.6	65.7 ± 0.4	22.5 ± 1.8	2.3 ± 0.14	101 ± 4.2

Data are presented as mean ± standard error mean (SEM). The differences between groups, pimobendan solution (PS) formulation and reference solution (RS) formulation (at the same timepoint and among timepoints within each group), were compared using two-way ANOVA with a repeated measure; values of *p* < 0.05 were considered significant. LA/Ao: left atrial to aortic ratio; LVIDDN: left ventricular internal diameter diastole normalize; LVIDSN: left ventricular internal diameter systole normalize; EF: ejection fraction; EDVI: end-diastolic volume index (corrected for the body surface area); ESVI: end-systolic volume index (corrected for the body surface area); SV: stroke volume; CO: cardiac volume; HR: heart rate. * Indicates significantly different from baseline of each group.

## Data Availability

The data that support the finding of this study are available from the corresponding authors (Anusak Kijtawornrat and Doungdaw Chantasart), upon reasonable request.
